# Nanotechnological approaches for management of soil-borne plant pathogens

**DOI:** 10.3389/fpls.2023.1136233

**Published:** 2023-02-17

**Authors:** Pranab Dutta, Arti Kumari, Madhusmita Mahanta, Gunadhya Kr Upamanya, Punabati Heisnam, Sarodee Borua, Pranjal K. Kaman, A. K. Mishra, Meenakshi Mallik, Gomathy Muthukrishnan, Kuttalingam G. Sabarinathan, Krishti Rekha Puzari, Dumpapenchala Vijayreddy

**Affiliations:** ^1^ School of Crop Protection, College of Post Graduate Studies in Agricultural Sciences, Central Agricultural University (Imphal), Imphal, India; ^2^ Sarat Chandra Singha (SCS) College of Agriculture, Assam Agricultural University, Dhubri, India; ^3^ College of Horticulture and Forestry, Central Agricultural University (Imphal), Pasighat, India; ^4^ Krishi Vigya Kendra (KVK)-Tinsukia, Assam Agricultural University, Tinsukia, India; ^5^ Department of Plant Pathology, Assam Agricultural University, Jorhat, Assam, India; ^6^ Department of Plant Pathology, Dr. Rajendra Prasad Central Agricultural University, Muzaffarpur, India; ^7^ Indian Council of Agricultural Research-National Centre for Integrated Pest management (ICAR-NCIPM), Pusa, New Delhi, India; ^8^ Agricultural College and Research Institute, Killikulam, Tamil Nadu Agricultural University (TNAU), Tuticorin, India

**Keywords:** nanotechnology, plant pathogen, plant health management, nanoformulation, mode of action

## Abstract

Soil borne pathogens are significant contributor of plant yield loss globally. The constraints in early diagnosis, wide host range, longer persistence in soil makes their management cumbersome and difficult. Therefore, it is crucial to devise innovative and effective management strategy to combat the losses caused by soil borne diseases. The use of chemical pesticides is the mainstay of current plant disease management practices that potentially cause ecological imbalance. Nanotechnology presents a suitable alternative to overcome the challenges associated with diagnosis and management of soil-borne plant pathogens. This review explores the use of nanotechnology for the management of soil-borne diseases using a variety of strategies, such as nanoparticles acting as a protectant, as carriers of actives like pesticides, fertilizers, antimicrobials, and microbes or by promoting plant growth and development. Nanotechnology can also be used for precise and accurate detection of soil-borne pathogens for devising efficient management strategy. The unique physico-chemical properties of nanoparticles allow greater penetration and interaction with biological membrane thereby increasing its efficacy and releasability. However, the nanoscience specifically agricultural nanotechnology is still in its toddler stage and to realize its full potential, extensive field trials, utilization of pest crop host system and toxicological studies are essential to tackle the fundamental queries associated with development of commercial nano-formulations.

## 1 Introduction

Soil is a reservoir of millions of microorganisms which imparts great impact on agriculture. A majority of microbes are beneficial for soil and plant health. However, some microorganisms pose great threat to crops as they often damage the root and crown tissues of plants thereby causing huge economic loses. Thus, pathogens which persist in the soil matrix or in residues over the soil surface are known as soil-borne plant pathogens (Veena et al., 2014). The soil-borne plant pathogens are distributed widely in soil however, few species exhibit localized distribution pattern. Soil-borne diseases caused by fungi, bacteria, nematodes, oomycetes, protozoa, viruses are considered vital in realization of potential yield in agricultural crops. Once established, these pathogens accumulate through synergistic associations and cause greater economic losses that are difficult to control. The soil-borne plant pathogens *viz*., *Fusarium* spp., *Rhizoctonia* spp., *Pythium* spp., *Sclerotinia* spp., *Verticillium* spp., and *Phytophthora* spp. can cause yield loss upto 50-75% for economically important crops such as wheat, maize, cotton, vegetables and fruits (Mihajlovic et al., 2017). *Fusarium oxysporum* strains alone can infect more than 150 agricultural crop species such as banana, tomato, melon, cotton etc. causing severe vascular wilt disease (Bertoldo et al., 2014). In cucurbitaceous crops, the pathogen is responsible for causing yield losses of around 30-80% (Lü et al., 2011). Fusarium wilt of banana, caused by *F. oxysporum* f. sp. *cubense* is a major threat to banana cultivation worldwide. The race Tropical Race 4 has been causing serious losses in Southeast Asian countries, thereby affecting the lives of small producers. Asides from wilt disease, some other strains of *Fusarium oxysporum* are capable of causing root/foot rot and damping off (Michielse and Rep, 2009). *F. solani* is mainly known to cause collar and root rots in many economically important crops such as beans and peas. *Fusarium* spp. also contaminate cereals and food grains by producing mycotoxins such as fumonisins, trichothecenes, zearalenone, and deoxynivalenol (Nelson et al., 1981). *F. graminearum* and *F. verticillioides* cause cob rot in maize, both species are known to produce mycotoxins. In oil seed rape (*Brassica napus*), the predominant population of *Rhizoctonia solani* AG2-1 isolate causes severe seedling diseases, establishment losses of up to 80-100%, and final yield losses of up to 30%. (Tahvonen et al., 1984; Kataria and Verma, 1992; Khangura et al., 1999). Rhizoctonia produces a variety of symptoms such as stem lesions, damping off, crown rot, root rot, stem rot and aerial web blight. The infection ultimately causes wilting, stunting and finally the death of the plant. The species of Phytophthora and Pythium cause damping off and root rot disease under cool and wet conditions and can affect 5-80% of the seedlings thereby incurring huge economic loss to the farmers (Alcala et al., 2016). Late blight caused by *Phytophthora* spp. is one of the most destructive soil-borne diseases of potatoes and tomatoes worldwide (Son et al., 2008). Worldwide, it causes an estimated loss of $5 billion annually (Latijnhouwers et al., 2004). Among the bacterial soil-borne pathogens, *Ralstonia solanacearum* causing bacterial wilt disease in more than 180 plants of 45 families ranks the first (Tahat and Sijam, 2010). In tomato crop, *R. solanacearum* can cause yield loss of 0-90% depending on the strain of the pathogen, cropping pattern, cultivar and climate (Nion and Toyota, 2015). Root knot nematodes (RKN) represent an important class of soil-borne pathogen that infect more than 5,500 host plants. *Meloidogyne* spp. are polyphagous, obligate sedentary, parthenogenetic and considered the most important plant parasitic nematode group worldwide (Jones et al., 2013). The typical symptoms produced by RKN include gall formation and damage to root system, along with above ground symptoms such as chlorosis, stunting, wilting and yield reduction (Karssen et al., 2013). The effect of RKN on the host plant is further intensified by the attack of secondary plant pathogens such root rot, fungal and bacterial wilt causing pathogens (Back et al., 2002; Karssen et al., 2013). The soil-borne diseases remain unnoticed until the above ground plant parts exhibit symptoms such as chlorosis, stunting, wilting and finally death. The common soil-borne diseases include damping off, root rot, vascular wilt etc. (Hornby et al., 1988). These diseases are often difficult to manage as they have wide host range and can survive for long periods on soil organic matter and plant debris, as free-living organisms or by producing resistant structures like sclerotia, microsclerotia, oospores or chlamydospore even in absence of host plant. Also, its diagnosis is difficult and cumbersome due to similarity in symptoms such as root rot, stunting, chlorosis, seedling damping, root blackening, bark cracking and branch and twig dieback (Patil et al., 2021). The non-specific symptoms and its resemblance with physiological disorders and water stress symptoms makes its timely diagnosis difficult (Åström and Gerhardson, 1988). Thus, the major hinderance in management of soil-borne diseases is its heterogenous incidence and scarce knowledge on the epidemiological aspects of pathogens. The experiences and observations passed on through several generations have given rise to cultural practices that reduce the losses caused by soil-borne plant pathogens but its effective management strategy still need to be explored. The expanding diversity of crops in agriculture emphasizes parallel expansion of strategies and develop novel strategies for effective management of soil-borne plant pathogens.

The farmers use synthetic fumigants and chemical fungicides at regular interval throughout the cropping season to minimize the soil-borne disease outbreak. However, extensive use of fumigants such as methyl bromide and fungicides disrupt the ecological balance, cause human and animal health hazards, damage to aquatic ecosystem and beneficial organisms in soil (Panth et al., 2020). The cultural practices such as crop rotation, biofumigation, anaerobic soil disinfestation, soil solarization, soil steam sterilization are mainly adopted by farmers to minimize the losses but these methods give inconsistent results and are less effective than chemical control methods. The mounting environmental constraints and ineffective management options emphasizes need of alternative sustainable and effective management strategy.

**Figure 1:**
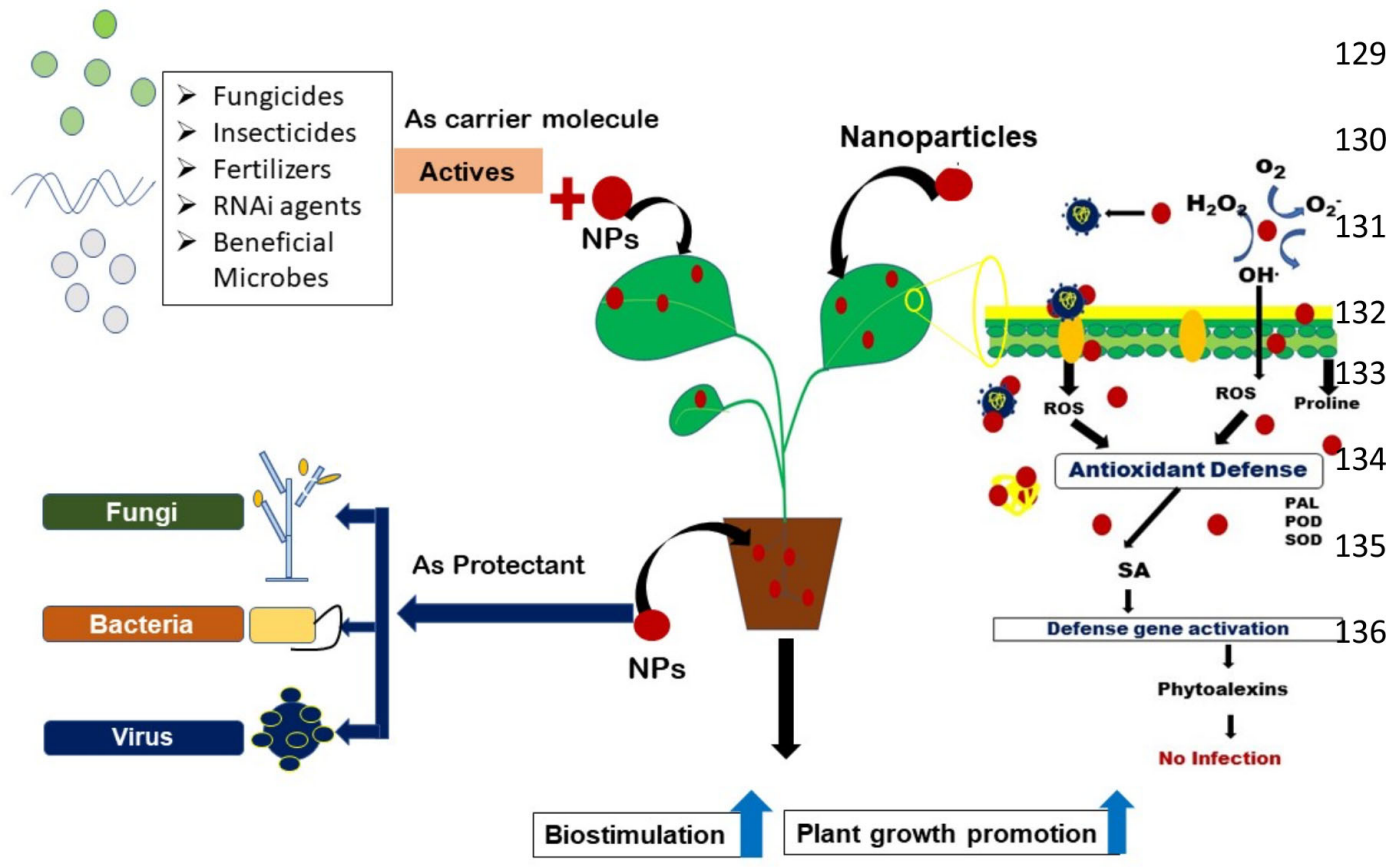
Schematic representation of different approaches of nanoparticles for management of soil borne plant pathogens.

Nanotechnology emerged as one of the most rapidly advancing science of twenty first century. Diversified application of nanotechnology in various fields have been found to uplift the entire scenario of industry and agricultural sector including information technology, medicine, disease detection and diagnosis, food safety and security, pest and disease management, environmental science and many more. From agriculture point of view, the major concern related to soil and environmental health includes: increased pesticide residue in soil and water bodies, decline in soil beneficial organisms, alteration of soil physical and chemical properties, pesticide resistance in pathogens and many more (Sharma et al., 2019). Nanotechnology can address most of these concerns and can bring revolutionary changes. It possesses marvelous application as antimicrobial and therapeutic compounds, targeted drug delivery, high sensitivity disease detection and diagnosis and thus likely to enhance agricultural productivity due to decline in cost associated with agricultural production practices (Dutta et al., 2021; Dutta et al., 2022). The small size of nanoparticles (<100 nm), greater surface area to volume ratio and high reactivity favors its wide-scale application in the field of human and plant pathology (Jeevanandam et al., 2018). The use of nano-encapsulated fertilizers and pesticides can reduce the amount of chemical fumigants and pesticides reaching the soil surface as compared to conventional formulations and also prolongs protection to plants against various phytopathogens. Nano-based materials can also act as cargo molecule and can release the active ingredients owing to its greater surface area to volume ratio. The effect on non-target organisms can also be reduced as they are highly target specific (Din et al., 2017). The disease tolerance ability of plants can also be enhanced thereby improving plant health (**Figure 1**). Thus, it may be predicted that integrity between timely and accurate disease diagnosis and management can be established in near future by exploiting the science of nanotechnology (Mahmood et al., 2017). Recent studies have revealed that nanoparticles show promising results as potential antimicrobial agent and biosensor for detection of plant pathogens especially against soil-borne plant pathogens. This review, focuses on all aspects of nanotechnology for management of soil-borne plant pathogens, thereby condensing scattered literature together at one place.

## 2 Nanotechnology in agriculture

In the field of agriculture, nanoscience is explored in delivery of plant hormones, seed germination, transfer of genes of interest, water management, nano-biosensors, nanobarcoding and controlled release of agro-chemicals (Hayles et al., 2017). Furthermore, nanoparticles are engineered with desired properties (i.e., size, shape, surface area etc.) for its use as protectant, therapeutant or site specific delivery of active ingredients such as fungicides *via* conjugation, adsorption or encapsulation (Khandelwal et al., 2016). The nano-based materials can be applied to plants as seed treatment, root dip treatment, soil application and foliar spray. The metallic oxides, nonmetals, metalloids, polymeric and carbon nanomaterials exhibit disease suppressing and growth promoting activities in plants (Elmer et al., 2018). The most popularly explored nanoparticles include silver, gold, copper, zinc oxide, iron and many more. The three main mechanisms involved in use of nanoparticles include: (a) nanoparticle as biosensor, (b) nanoparticle as protectant or therapeutant, and (c) nanoparticle as smart delivery vehicle of fungicides or actives such as target genes. Nanoparticles such as quantum dots and metallic nanoparticles can be functionalized with biological markers for *in situ* and rapid detection of soil-borne pathogens. The nanoparticles have the potential to serve as protective or therapeutic agents against a variety of soil-borne pathogens, *viz*., *Fusarium oxysporum, Sclerotium rolfsii, Rhizoctonia solani, Sclerotinia sclerotiorum* (Kaman and Dutta, 2019; Abdelrhim et al., 2021) *Ralstonia solanacearum* (Khairy et al., 2022), soil-borne viruses *viz*., Barley yellow mosaic virus (BaYMV) (Aref et al., 2012), etc. The main mechanism of action against microorganisms include agglutination and cell membrane disruption, inhibition of synthesis of RNA, proteins, toxins, enzymes such as H+-ATPase and blockage of flow of nutrients (Dakal et al., 2016; Malerba and Cerana, 2016). Nanoparticles also acts as carrier molecule and allow target-specific release of active ingredients into the plant system, thereby reducing the load of chemicals into the environment. The nano-based formulations provide several benefits such as improved water solubility of pesticides, site specific delivery and uptake by target sites, increased shelf life, reduced effect on non-target organisms and residual effect on environment (Hayles et al., 2017). Also, the stability and activity of nano-based formulations are greater as compared to conventional pesticides even under unfavorable environmental conditions (rainfall and UV exposure), thereby reducing the number of applications, toxicity and overall costs.

Another important aspect of plant health management is the use of fertilizers. Nanofertilizers bear the potential to increase the release and uptake efficacy of nutrients thereby boosting plant disease resistance. It has widely been explored in plant/disease systems *viz*., Fusarium wilt in tomato, chrysanthemum, root and crown rot in asparagus, red root rot in tea, verticillium wilt in brinjal (Elmer et al., 2018).

## 3 Nanotechnology based diagnosis of soilborne pathogens

Rapid detection and diagnosis of soil-borne pathogens is fundamental for its effective and timely management. A number of immunological, serological, nucleic acid-based detection assays have been developed for accurate detection of plant pathogens. Over the past two decades, numerous efforts have been made to develop methods for diagnosing and monitoring plant infections using biochemical assays utilizing specific proteins, toxins, ELISA, nucleic acid probe technologies, and PCR amplification of nucleic acid sequences (McCartney et al., 2003; Sundelin et al., 2009; Kashyap et al., 2013a; Kumar et al., 2013; Kumar and Kashyap, 2013; Singh et al., 2014). These biochemical assays and nucleic acid-based methods are sensitive, exact, and useful for verifying visual scouting, but they are unsuitable as screening tests to check on the health of plants before symptoms manifest. They necessitate intricate sampling techniques, costly infrastructure, and might mask the true state of pathogen infections. Unfortunately, only a small number of plant diseases can be effectively detected using these assays. However, the majority of these techniques are ineffective for on-site disease detection in crop fields. The use of molecular approaches is further constrained by the high cost and limited shelf life of molecular biology reagents like enzymes and primers. Therefore, the introduction of low-cost techniques to increase the precision and speed of plant pathogen diagnostics is required.

Recent advancement in nanotechnology has led to development of functional nanoparticles (electronic, optical, magnetic, or structural) which can be covalently attached to biological molecules including nucleic acids, peptides and proteins. Quantum dots (QD), one of the most promising nanomaterials, have been extensively exploited in a wide range of bio-related applications, including the quick and precise detection of a specific biological marker (Kashyap et al., 2015).Tools for high-throughput analysis, high-quality monitoring, and crop protection such as biosensors, quantum dots, nanostructured platforms, nanoimaging, and nanopore DNA sequencing have the potential to increase the sensitivity, specificity, and speed of disease detection (Khiyami et al., 2014). Additionally, nano-diagnostic kit tools are rapid and simple to use in identifying potential plant pathogens, enabling specialists to assist farmers in the prevention of epidemic illness. The ability of QD-based nanosensors to simultaneously probe several enzyme activities has been demonstrated by Knudsen et al. (2013). CdTe quantum dots have been utilized as biosensors by coating with specific antibodies against the glutathione S-transferase (GST) protein of *Polymyxa betae*, the vector of BNYVV causing rhizomania disease in sugar beet (Safarpour et al., 2012). In order to detect the harmful fungus *Sclerotinia sclerotiorum*, Wang et al. (2010) used indirect stimulation to construct a sensitive electrochemical sensor employing a modified gold electrode with copper nanoparticles. They used this sensor to successfully and precisely quantify salicylic acid in oilseeds to detect the pathogen. It is necessary to conduct more research on related sensors and sensing systems to detect pathogens, their byproducts, or to track physiological changes brought on by infections in plants. Schwenkbier et al. (2015) created a helicase-dependent isothermal amplification (HDA) in conjunction with on-chip hybridization for identifying *Phytophthora* species. With this method, the target gene locus for the yeast GTP-binding protein (Ypt1) can be amplified effectively at a single consistent temperature in a tiny heating unit. By using on-chip DNA hybridization and subsequent silver nanoparticle deposition, the assay’s specificity was established. The silver deposits act as reliable endpoint signals, enabling both electrical and optical readout. These developments suggest that the combined techniques will soon be applied on-site for the accurate identification of several soil-borne pathogens. Hervas et al. (2011) established a “lab-on-chip” method for the rapid, sensitive, and selective quantification of zearalenone generated by *Fusarium* sp. that incorporates an electrokinetic magnetic bead-based electrochemical immunoassay on a microfluidic chip. Rispail et al. (2014) studied the effects of superparamagnetic nanoparticles and quantum dots on *Fusarium oxysporum*. The presence of the pathogenic fungus was quickly identified by interactions between nanomaterials and the fungal hypha, though their internalization patterns varied. This study showed viability of new nanotechnology-based systems for the early detection and eventual control of harmful fungi which is the first study on the effects of quantum dots and superparamagnetic particles on fungal cells. Hashimoto et al. (2008) created a novel biosensor system comprising two biosensors for rapid detection of soil-borne pathogens. Here, equal amounts of two distinct microorganisms, each immobilized on an electrode, were used to build the system.

However, the science of nanodiagnostics is still in the toddler stage for accurate detection of pathogens and toxins in agricultural field. Extensive research is needed to optimize the diagnostic assays for detection of precise signals emitted from low level of pathogens. Also, efforts must be channelized towards development of portable, cheap, efficient and hand held nanodevices for *in situ* detection of soil borne pathogens.

## 4 Nanotechnology for management of soil-borne pathogens

### 4.1 Nanoparticles as protectants

Nanoparticles alone can be directly utilized as antimicrobial agent and have been found effective against numerous soil-borne pathogens. It can be applied to soil, seed, root, foliage for providing protection against pests and pathogens such as fungi, bacteria and viruses. Nanoparticles penetrate the plant system and directly acts against the pathogen or it behaves as elicitor molecule for inducing local and systemic defense responses in plants. Metallic nanoparticles such as gold, silver, titanium-oxide, zinc-oxide, copper oxide are most intensely studied nanoparticles and known to exhibit antifungal, antibacterial and antiviral properties (Gogos et al., 2012; Kah and Hofmann, 2014; Kim et al., 2018). Several studies reported nanoparticles as an effective antimicrobial agent to curb the menace caused by soil-borne phytopathogens.

Kaman and Dutta (2019) studied the antifungal activity of biogenically synthesized silver nanoparticles (AgNPs) against soil-borne phytopathogens *viz*., *Sclerotium rolfsii*, *Rhizoctonia solani*, *Fusarium oxysporum* and *Sclerotinia sclerotiorum* at 100 ppm AgNP concentration. Desai et al. (2021) reported the antifungal activity of AgNPs against the pathogen *Sclerotium rolfsii* in wheat plant. They observed 100 per cent mycelial growth inhibition and sclerotial germination inhibition under both *in vivo* and *in vitro* condition at 100 ppm AgNP concentration. However, the root of wheat plants exhibited phytotoxic effect at this concentration. Thus, 50 ppm AgNP concentration was inferred best in terms of disease management and plant growth. Another study conducted by Zaki et al. (2022) revealed antifungal activity of mycogenically synthesized Zinc oxide nanoparticles (ZnONPs) mediating *Trichoderma* spp., against soil-borne pathogens *viz*., *Rhizoctonia solani, Macrophomina phseolina* and *Fusarium fujikuroi*). Significant antifungal effect was recorded under *in vitro* condition as well as on cotton seedlings. Also, inhibitory effect of AgNPs was reported in a dose dependent manner against mycelial growth of *R. solani*, *S. sclerotiorum* and *S. minor* (Min et al., 2009). The mode of action of AgNPs as revealed from microscopic observations indicates fungal cell disintegration, separation of layers of hyphal wall and ultimately hyphal collapse and death. Tomah et al. (2020) determined the antifungal activity of AgNPs synthesized mediating *Trichoderma* spp. against *Sclerotinia sclerotiorum. In vitro* antifungal assays reported 93.8%, 100% and 100% inhibition of sclerotial formation, myceliogenic germination and hyphal growth at 200 µg/mL AgNP concentration respectively. The SEM and EDS study indicated direct interaction of nanoparticles and fungal cells including AgNP contact and accumulation within fungal cells, micropore or fissure formation on fungal cell wall and lamellar fragment production. Similar results were obtained by Guilger-Casagrande et al. (2021) against *Sclerotinia sclerotiorum.* Chen et al. (2020) evaluated the effect of Magnesium oxide nanoparticles (MgONPs) against *Thielaviopsis basicola* and *Phytophthora nicotianae. In vitro* studies revealed inhibition of fungal growth, spore germination and impediment of sporangium development. Direct interaction, adsorption of nanoparticles by fungal hypha and cell morphological changes were the underlying mechanism involved in antifungal effect as confirmed by SEM, TEM and EDS. Under greenhouse conditions, 42.35% and 36.58% decline in tobacco black root rot and black shank disease respectively was observed at 500 µg/ml of MgONP testifying suppression of fungal invasion through root irrigation. Juan-ni et al. (2022) observed similar results for copper oxide nanoparticles (CuONPs) against *Phytophthora nicotianae.* They observed 33.69% increase in control efficacy and tobacco black shank disease suppression without inducing phytotoxicity at 100 mg L^−1^ of CuONPs treatment under pot condition. Additionally, they reported increased SOD enzyme activity and intracellular ROS accumulation as antifungal mechanisms of the used nanoparticles. Exposure of tobacco plants to CuONPs also significantly activated cascade of defense enzymes, resistance genes and Cu-content in leaves and root of treated plants. Encinas et al. (2020) showed silver-chitosan nanoparticles significantly inhibited mycelial growth of *Fusarium oxysporum* upto 70% and reduced the severity of the disease in *Fusarium oxysporum* inoculated tomato seedlings after 14 days post inoculation. Further, nanoparticles did not exhibit any negative impact on vegetative development of the seedlings upto 2000 ppm concentration.

Jiang et al. (2021) tested three metal oxide NPs *viz*., ZnO, FeO and CuO NPs against the tomato bacterial wilt pathogen *Ralstonia solanacearum*. The results showed nanoparticles especially CuONPs significantly reduced incidence of tomato bacterial wilt disease caused by soil-borne bacterium *Ralstonia solanacearum*. Also, significant improvement in morpho-physiological parameters of infected plants, diversity and richness of rhizospheric bacterial community were observed (**Table 1**).

**Table 1:** Use of Nanoparticles for management of soil-borne plant pathogens.

Nanomaterial	Type of Pathogen	Target Pathogen	Crop	Effect	Reference
As Protectant					
Silver Nanoparticles (AgNPs)	Fungi	*Rhizoctonia solani, Fusarium oxysporum, Sclerotium rolfsii* and *Sclerotinia sclerotiorum*	Cereals, Pulses and vegetables	Mycelial growth inhibition at 100 ppm AgNP	Kaman and Dutta (2019)
AgNPs	Fungi	*Fusarium fujikuroi, Rhizoctonia solani and Macrophomina phseolina*	Cotton (*Gossypium herbaceum)*	Reduction in mycelial growth and illness of cotton seedlings	Zaki et al. (2022)
AgNPs	Fungi	*Sclerotinia sclerotiorum*	Mustard (*Brassica juncea*)	Inhibition of hyphal growth, sclerotial formation and myceliogenic germination of sclerotia	Tomah et al. (2020)
Capped AgNPs	Fungi	*Sclerotinia sclerotiorum*	Vegetables	Inhibition of mycelial growth and sclerotia germination	Guilger-Casagrande et al. (2021)
Magnesium oxide nanoparticles (MgONPs)	Fungi	*Thielaviopsis basicola* and *Phytophthora nicotianae*	Tobacco (*Nicotiana tabacum)*	Inhibition of fungal growth, spore germination and impediment of sporangium development	Chen et al. (2020)
MgONPs	Fungi	*Phytophthora infestans*	Potato (*Solanum tuberosum*)	Inhibition of *Phytophthora infestans* by cell membrane distortion, oxidative stress, disruption of metabolic pathways and membrane transport activity with no harmful effect on potato	Wang et al. (2022)
Copper oxide nanoparticles (CuONPs)	Fungi	*Phytophthora nicotianae*	Tobacco *(Nicotiana tabacum)*	33.69% increase in control efficacy and tobacco black shank disease suppression without inducing phytotoxicity at 100 mg L^−1^ of CuO NPs treatment	Juan-ni et al. (2022)
Chitosan NPs	Fungi	*Rhizoctonia solani, Fusarium oxysporum, Sclerotium rolfsii*	Cereals, Vegetables	Reduction in mycelial growth	Boruah and Dutta, 2021
Carboxymethyl cellulose coated core/shell SiO_2_@Cu nanoparticles	Fungi	*Phytophthora capsici* Host: Black pepper	Black pepper (*Piper nigrum)*	Antifungal activity against *P. capsici* with MIC 75 ppm	Hai et al. (2021)
AgNPs	Fungi	*Macrophomina phaseolina* and *Fusarium solani*	Strawberry (*Fragaria ananassa)*	The nanoparticle showed broad spectrum antagonism against *M phaseolina* (67.05%) and *F. solani* (83.05%)	Paola et al. (2018)
AgNPs	Fungi	*Phomopsis* sp. Host: Soybean seeds	Soybean (*Glycine max)*	Absolute inhibition of the pathogen was observed 270 and 540 ppm concentration	Mendes et al. (2014)
Zinc oxide (ZnO), Iron oxide (FeO) and Copper oxide (CuO) nanoparticles	Bacteria	*Ralstonia solanacearum* Host: Tomato	Tomato *(Solanum lycopersicum)*	Reduced incidence of tomato bacterial wilt disease	Jiang et al. (2021)
Gold nanoparticles (AuNPs)	Virus	Barley yellow dwarf virus (BaYDV)	Barley (*Hordeum vulgare*)	A high yield of ruined virus like particles (VLPs) were also observed	Alkubaisi and Aref (2016)
Copper/Iron NPs	Nematodes	*Meloidogyne incognita* and *M. javanica*	Tomato *(Solanum lycopersicum)*	Nematicidal activity such as paralysis, biological cycle arrest, reduction in number of galls with lowest EC50 value as compared to commercial nematicides	Gkanatsiou et al. (2019)
AgNPs	Nematodes	*Meloidogyne javanica*	Tomato *(Solanum lycopersicum)*	Nematicidal activity on egg hatchability and juvenile mortality. Reduction in number of galls, egg masses, number of females per root/plant and mortality of juveniles.	Ghareeb et al. (2020)
As Carrier of Actives					
*Pectobacterium cypripedii* nanoghost loaded with tebuconazole	Fungi	*Leptosphaeria nodorum, Pyrenophora teres*	Barley (*Hordeum vulgare*) and Wheat (*Triticum aestivum*)	The efficacy of the loaded bacterial ghost for resistance to rainfall and the protective and curative effects against the pathogens increased	Hatfaludi et al. (2004)
Lignin-modified polymer nanocapsule loaded with pyraclostrobin	Fungi	*Fusarium. oxysporum* f. sp. *radicis-lycopersici*	Tomato *(Solanum lycopersicum)*	The nanocapsules lead to rapid release of actives and increased its efficacy against the pathogen and soil mobility. Also, residue development in soil was reduced	Luo et al. (2020)
Carbendazim-loaded polymeric nanoparticles	Fungi	*Fusarium oxysporum* and *Aspergillus parasiticus*	*Cucumber (Cucumis sativa), Maize (Zea mays)* and Tomato *(Solanum lycopersicum)*	Increased rate of fungal inhibition	Kumar et al. (2017)
lecithin/chitosan-encapsulated kaempferol	Fungi	*Fusarium oxysporum*	Vegetables and stored food products	67% inhibitory efficiency after 60 days of storage on a Petri dish with *Fusarium oxysporum*-infected fungus	Ilk et al. (2017)
Combination of AgNPs and Fluconazole	Fungi	*Phoma glomerata, Phoma herbarum, Fusarium semitectum, Trichoderma* sp.*, and candida albicans*	Pulses, Vegetables	Enhanced antifungal activity of fluconazole against *Phoma glomerata, Trichoderma* sp.*, and candida albicans*	Gajbhiye et al. (2009)
SLNs loaded with essential oil of *Zataria multiflora*	Fungi	*Rhizoctonia solani*	Cereals, Pulses, Post-harvest pathogens	Stabilization of essential oil of *Zataria multiflora*, thereby increasing its efficacy	Nasseri et al., 2016
Alginate-gelatin nanocomposite encapsulated *Pseudomonas fluorescens* (VUPF5 and T17-4 strains)	Fungi	*Fusarium solani* Crop: Potato	Potato (*Solanum tuberosum*)	The encapsulated *Pseudomonas fluorescence* strains showed enhanced shelf life than non-coated bacteria. Also, the green house experiment revealed increased control efficacy against *Fusarium solani* causing disease in potato plants.	Pour et al. (2019)
*T. asperellum* and nanochitosan based formulation	Fungi	*Rhizoctonia solani, Fusarium oxysporum, Sclerotium rolfsii*	Cereals, Pulses, Vegetables	Enhanced reduction in mycelial growth of the pathogens	Boruah and Dutta (2021)
Encapsulated *Streptomyces fulvissimus* Uts22 strain based on alginate–Arabic gum and nanoparticles (SiO_2_ and TiO_2_)	Fungi	*Pythium aphanidermatum* Crop: Cucumber	Cucumber (*Cucumis sativus*)	Encapsulated bacteria resulted in a 95% reduction in damping-off disease of cucumber and showed more potential effects on increasing plant growth traits than free bacteria under green-house condition.	Saberi Riseh et al. (2022)
*Bacillus subtilis* Vru 1 encapsulated in alginate-bentonite coating and enriched with titanium nanoparticles	Fungi	*Rhizoctonia solani* Crop: Bean	Bean (*Phaseolus vulgaris* L.)	Vru 1 nanocapsules showed 90% inhibition of the pathogen as compared to 60% inhibition by free Vru 1. Also, vegetative growth parameters in bean plant were significantly enhanced	Saberi-Rise and Moradi-Pour (2020)
Nanoparticles as stimulator of plant growth and development					
AgNPs	Fungi	*Rhizoctonia solani* Crop: Rice	Rice (*Oryza sativa*)	Inhibition of mycelial growth and sclerotial germination Reduction in per cent disease incidence, enhanced plant growth parameters and secondary metabolites *viz*., phenols, flavonoids, terpenoids and TSS	Dutta et al. (2021)
Silicon dioxide nanoparticles (SiO_2_ NPs)	Fungi	*Rhizoctonia solani*	Wheat (*Triticum aestivum*)	Increased the amount of chlorophylls, carotenoids, defense-related stimulants (particularly salicylic acid), POD, SOD, APX, CAT, and PPO enzymes, phenolics and flavonoids antioxidant defense mechanisms.	Abdelrhim et al. (2021)
ZnO-NPs	Fungi	*Fusarium oxysporum*	Brinjal (*Solanum melongena*)	Increased plant height, root length, plant fresh biomass, chlorophyll a, chlorophyll b, total soluble carbohydrates, total soluble protein, phenol, antioxidant activity, and isozymes	Abdelaziz et al. (2022)

Alkubaisi and Aref (2016) studied the effect of gold nanoparticles (AuNPs) on soil-borne Barley yellow dwarf virus (BaYDV) using TEM. They observed that dual existence of AuNPs *in vivo* and *in vitro* affected the configuration of capsid protein of virus after 24 and 48 hours of incubation period. Also, the size of nanoparticles plays critical role in reducing virus infectivity. The AuNPs of size 3.151 and 31.67 nm caused deterioration of virus particle by 75.3% and 24.7% respectively. A high yield of ruined virus like particles (VLPs) were observed in the local cultivar *Hordeum vulgare*. After 48 hours, completely lysed VLPs and some deteriorated VLPs were observed.

The increased potential and application of metallic nanoparticles indicates greater exposure of biological systems to metallic nanoparticles. Thus, understanding the interaction of metallic nanoparticles with plants, animals, human as well as environment is of utmost importance. Natural nanoparticles are constantly present in the environment, thus relationship between nature and nanoparticles is quite ancient. Biological beings have evolved both genetically and phenotypically under different types of stress conditions and possess defense mechanisms to counteract these adversities. However, engineered nanoparticles presents new concern to the ecological balance. The heavy metal nanoparticle mediated stress in plants leads to generation of variety of reactive oxygen species (ROS) which induce different responses in plants such as oxidative damage, lipid peroxidation, alteration in ion transport across cell membrane, malfunctioning of mitochondrial DNA, proteins and chloroplast. Plants have developed mechanisms to defuse these radicals however, balance between ROS generation and detoxification is essential for protection of plants. Gopalakrishnan Nair et al. (2014) reported increase in hydrogen peroxide content and lipid peroxidation in Mung bean plant exposed to CuONPs. Another study reported iron oxide phytotoxicity in *Lemna minor* plant which caused enhanced production of ROS and malondialdehyde in a dose-dependent manner (Souza et al., 2019). Plants also possess ROS scavenging mechanism that aids plants in overcoming these stresses (Czarnocka and Karpiński, 2018). The antioxidant enzymes include superoxide dismutase (SOD), catalase, glutathione peroxidase, glutathione reductase, ascorbate peroxidase etc. The antioxidant mechanism and ROS production depends on plant species, concentration, type of nanoparticles and the duration of exposure. Several studies as discussed in later part of the article reported increase in radical scavenging enzymes in nanoparticle treated plants which in turn enhances plant growth, development and yield. Thus, the relationship between plant adaptability and phytotoxicity is still debatable and needs further research to gain deeper insights. Another major concern is the accumulation of metallic nanoparticles in ecosystem that presents continuous threat to human and ecological health. Metallic nanoparticles are known to interact with cell membrane, damage membrane permeability, DNA, proteins and can easily enter the into the bloodstream and accumulate into the vital organs thereby causing toxicity (Hsin et al., 2008). The nano-size allows particles to gets easily absorbed, 15-20 times greater than bulk counterparts into any system including biological systems. They also get absorbed in the soil, water and air ecosystem and may enter the food chain and deleteriously affect the natural fauna including beneficial organisms and microbes. Aquatic ecosystem is another habitat vulnerable to nanotoxicology due to accumulation of nanoparticles through surface run off. Several toxicity studies have been conducted on aquatic species but genotoxicity is not well established in these species. (Baalousha et al., 2011). Also, the engineered nanoparticles are analogous to heavy metal oxides and their behaviour and fate are affected by aggregation processes. Nanoparticles tends to aggregate and settle down leading to water decontamination due to loss of pollutants. On the other hand, nanoparticles have also been reported to exert toxicity on aquatic organisms including algae, plants, microorganisms, invertibrates and vertibrates (Zhang et al., 2018). Thus, the research on nanotoxicology have although increased in the recent decade however more studies need to be channelized towards this area to understand the environmental fate, transformation, bioavailability, transport, relevant toxicity and draw conclusive remark.

Polymeric nanoparticles such as cellulose, lignin and chitosan constitute important group of antimicrobial compounds possessing antifungal, antibacterial properties as well as plant growth promoting abilities. These compounds are considered as environment friendly as these are biodegradable and are abundantly found in nature. Dawwam et al. (2022) green synthesized cellulose nanocrystals (CNs) using agro-wastes obtained from palm sheath fibers and ZnONPs were synthesized using sono-co-precipitation method. The CNS-ZnO bio-nanocomposite were evaluated for antibacterial activity against gram negative (*Escherichia coli* and *Salmonella*) and Gram-positive (*Listeria monocytogenes* and *Staphylococcus aureus*) bacteria which possess ability to persist in soil. They observed MIC value of the CNS-ZnO nanocomposite were in the range 0.5-1.0 µg/ml against *E. coli* and *L. monocytogenes* while the MIC values against *Salmonella* and *S. aureus* were 0.25 – 1 µg/ml indicating influence of CNS-ZnO at low concentration. Also, the virulence and toxin associated genes of the bacterial pathogens were found to be downregulated suggesting anti-toxigenic properties of the CNS-ZnO nanocomposites. In another study, Schiavi et al. (2022) synthesized cellulose nanocrystals (CNCs) from the wastes obtained from olive pruning using chemical bleaching method. The synthesized nanocrystals exhibited inhibition of bacterial pathogen causing olive knot disease (*Pseudomonas savastanoi* pv. *savastanoi*) under *in vitro* condition. An inhibition of bacterial biofilm formation and reduction in bacterial epiphytic survival with no adverse effect on leaf development and root uptake were reported from their study. Lignin is another important aromatic polymer obtained from natural sources which possess antimicrobial activity and can be used as nanocarriers. Paul et al. (2021) synthesized lignin nanospheres using combination of solvent displacement method with sonochemistry and reported increased inhibition of Gram-positive *Bacillus megaterium* and gram-negative *E. coli.* Chitosan nanoparticles (ChNPs) evolved as a promising antimicrobial and plant growth promoting compound having potential to be used in management of soil-borne pathogens. The biopolymer based ChNPs were found effective against numerous pathogens *viz*., *Rhizoctonia solani* (Saharan et al., 2013; Boruah and Dutta, 2021), *Fusarium oxysporum* (Muthukrishnan and Ramalingam, 2016; Boruah and Dutta, 2021), *Sclerotium rolfsii* (Boruah and Dutta, 2021). Kheiri et al. (2016) reported synthesis of ChNPs of different molecular weight and demonstrated its antifungal activity against Fusarium head blight pathogen *Fusarium graminearum*. The per cent mycelial growth inhibition was recorded as 77% at 5000 ChNP concentration. The greenhouse trial indicated decline in AUPDC in treated plants. The ChNPs were also found effective against *Macrophomina phaseolina* and *R. solani* and showed inhibition of radial growth of the pathogen in a dose dependent manner (Saharan et al., 2013). Suarez-Fernandez et al. (2020) reported that root exudates from chitosan treated tomato plants inhibited soil borne fungal pathogen *F. oxysporum* f.sp. *radicislycopersici* and root knot nematode *Meloidogyne javanica*. Two-fold reduction in mycelial growth of fungal pathogen was observed with respect to control and 1.5-fold reduction in hatching of *M. javanica* eggs were recorded after 72 hours. Khairy et al. (2022) reported that ChNPs were effective against bacterial wilt of tomato and potato caused by *Ralstonia solanacearum*. *In vitro* assay indicated highest zone of inhibition at 200 μg/ml concentration. *In vivo* assay exhibited decline in disease incidence and severity after foliar application of ChNPs in wilt affected plants. The ChNPs were found to directly interact with bacterial cell wall causing alteration in shape, loss of flagella and ultimately cell lysis. The results obtained from RAPD-PCR revealed differences in genotype of exposed *Ralstonia solanacearum* as compared to untreated ones. Kim et al. (2013) synthesized Chitosan-lignosulfonate (CS-LS) nanohybrid and reported inhibition of *Bacillius subtillis, S. aureus* and *E. coli* at a rate higher than CS and LS alone.

**Figure 2:**
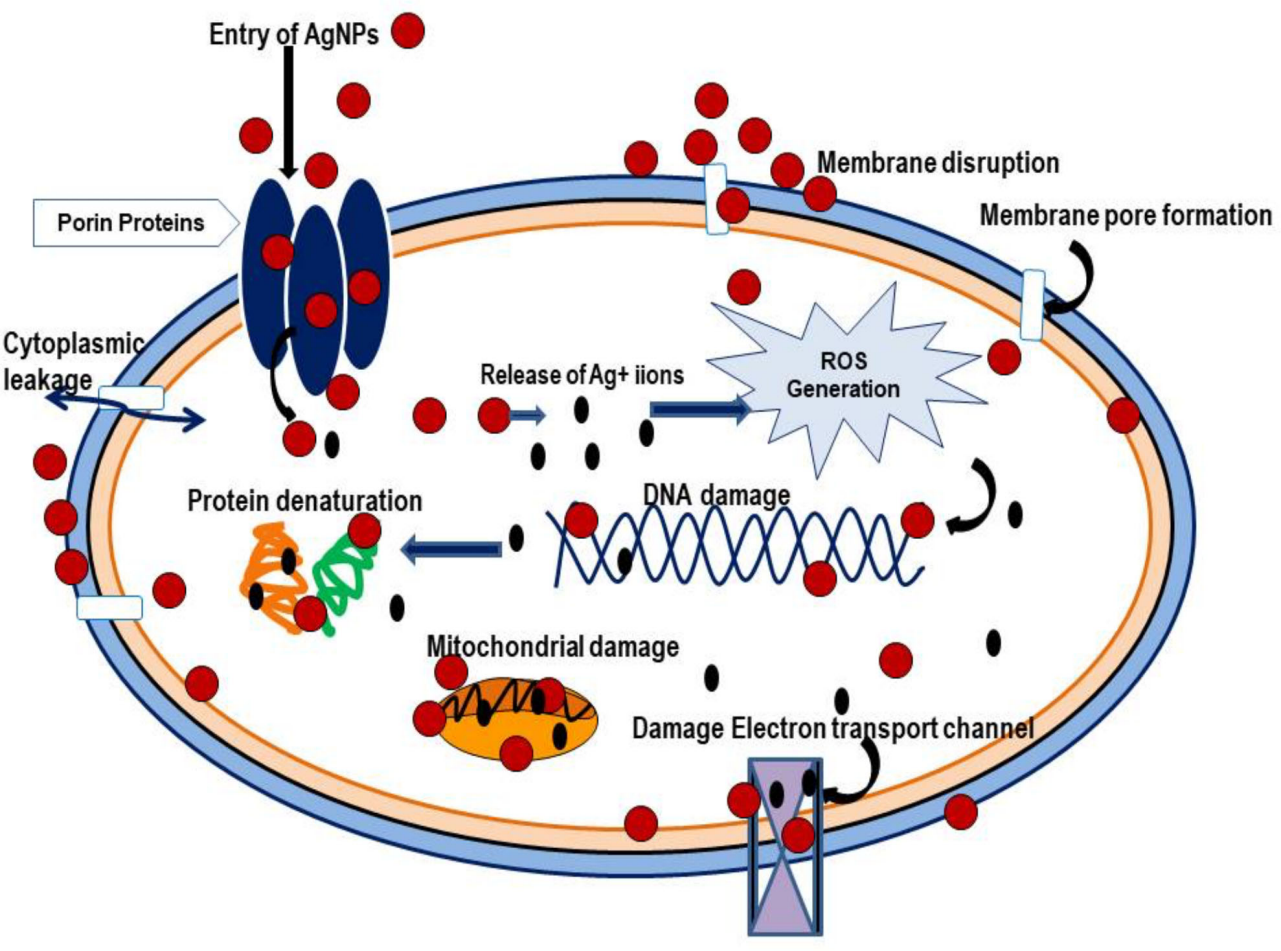
Mechanism of antimicrobial action of nanoparticles on Microorganisms.

From the above discussion, we can infer that nanoparticles have been widely studied for its antimicrobial activity against soil-borne pathogens more specifically fungal pathogens. The literature on effect of nanoparticles on soil-borne bacterial and viral pathogens is scarce. The metallic nanoparticles such as Ag, ZnO, and CuO NPs have been found to produce promising results in management of most of the soil-borne pathogens. The positively charged metallic nanoparticles can easily be adsorbed on the surface and penetrate into the cell as compared to its bulk counterparts (Gupta and Rai, 2017). Also, the mode of action of nanoparticles as established from previous studies include nanoparticle contact, accumulation, cell wall disruption, membrane leakage, inhibition of RNA and protein synthesis, inhibition of ATPase activity, intracellular ROS production and increased SOD activity (Dakal et al., 2016) (**Figure 2**). The antiviral activity includes direct interaction of nanoparticles with capsid protein and degradation of virus particle (Alkubaisi and Aref, 2016). More research is needed especially against soil-borne bacteria and viruses to determine its exact mode of action. Further, *in vivo* trials are needed to validate the results obtained from *in vitro* assays. However, the use of heavy metal nanoparticles raises toxicity concerns and its persistence and accumulation in the food chain as well as ecosystem. Extensive research is needed in this regard to obtain accurate conclusions. Natural polymeric nanoparticles such as chitosan, lignin and cellulose could prove as a suitable alternative as these are biodegradable and environment friendly in nature.

### 4.2 Nanomaterials as carrier of antimicrobial agents

Nanotechnology can meet the need of sustainable agriculture by reducing the load of chemical fungicides and pesticides in the environment. The nanoparticles as carrier molecule allows slow, timely and targeted release of active ingredients in the environment thereby increasing its efficacy. The soil-borne pathogens or its propagules do not invade the roots of the plant at the same time, thus persistent and slow release of active ingredients is essential to provide protection to the crop throughout the growing season (Khan et al., 2011). Nanocarriers reduce the rate of application as the chemical actually effective against the pathogen is 10-15% lesser than the chemicals required with conventional formulations. The nanomaterials having small size, greater kinetic stability, low viscosity, and optical transparency can prove better and smart delivery vehicle (Xu et al., 2010). The nanoformulations as carrier of active antimicrobial agents can enhance the solubility, wettability, dispersion and bioavailability of chemical fungicides and pesticides (Bergeson, 2010a). Nanomaterials exhibit unique properties such as solubility, thermal stability, permeability, crystallinity and biodegradability (Bordes et al., 2009; Bouwmeester et al., 2009) essential for nanopesticide development. The common nano-based delivery systems that potentially be effective in plant protection strategies include nanoencapsulates, nanotubes, nanowires, nanoemulsions and nanocages (Bouwmeester et al., 2009; Lyons and Scrinis, 2009; Bergeson, 2010b). Ghormade et al. (2011) summarized the studies on use of nano-based smart delivery systems for agrochemicals viz., pesticides, fertilizers and plant growth promoters. Nanoencapsulates, primarily nano-clay provides interactive surfaces having aspect ratio for encapsulation of agrochemicals (**Table 1**).

In 1997, the first research on nano-based fungicides was carried out, and efforts were made to embed fungicides into solid wood (Liu et al., 2001; Liu et al., 2002). Since then, numerous investigations using a variety of nanoparticles have been undertaken using regular fungicides (20 studies) and biocides possessing antifungal capabilities (6 studies). A variety of essential oils not covered by the fungicide groups were investigated, along with nine Fungicide Resistance Action Committee (FRAC) groups. Polymer blends, silica, and chitosan were the most frequently researched nanoparticle carriers. To assess the effectiveness of the nanofungicide, a variety of fungi were used. However, there weren’t many toxicity studies, nor were many plants studied. Nanosized bacterial ghosts, which are non-attenuated void cell envelopes of Gram-negative bacteria, were utilized by Hatfaludi et al. (2004) to improve the low water-solubility of tebuconazole and boost adhesion to the leaf surface. *Pectobacterium cypripedii* as the nano-bacterial ghost was chosen due of its capacity to stick to plants. When exposed to severe simulated rain under glasshouse settings, fluorescently labelled ghosts stuck to rice leaves the best (retaining 55%) and soya leaves the least (10%) of the six plants examined (rice, soya, cabbage, cotton, barley, and maize). All six plants were tested against a variety of fungi using ghost-loaded tebuconazole or two different commercial tebuconazole treatments (WP 25 and EW 250). Plants that weren’t exposed to rain had protection that was at par with or better than that of commercial treatments with one exception. None of the groups outperformed the commercial solutions when the plants were exposed to intense rain and fungus one hour after treatment except one. However, ghost-loaded tebuconazole was equivalent to, or larger than, WP 25 treatments when rinsed 24 hours after treatment, although EW 250-treated controls were often more effective. Luo et al. (2020) attempted to develop a nanoscale delivery system for low water soluble fungicide pyraclostrobin. The active ingredient was encapsulated using lignin-modified polymer nanocapsule to enhance soil mobility. The nanocapsules allowed rapid release of actives and increased the distribution and accumulation of active ingredient on the surface of target organisms thereby increasing its efficacy and also soil mobility. The pot trials revealed nanoemulsions in water improved control efficacy against tomato crown and root rot caused by *Fusarium. oxysporum* f. sp. *radicis-lycopersici* as compared with micron-grade microcapsule suspension (CS) of pyraclostrobin. Also, lower fungicide residue in soil was evident than CS treatment. Xu et al. (2014) conducted similar study where pyraclostrobin was encapsulated using chitosan–lactide copolymer nanoparticles. Initially, the nanofungicide was less efficient in inhibition of *C. gossypii* as compared with commercial pyraclostrobin. However, seven days post treatment, an increase in pathogen inhibition was observed greater than the active alone. In a different experiment, lecithin/chitosan-encapsulated kaempferol (another low-soluble fungicide) demonstrated 67% inhibitory efficiency after 60 days of storage on a Petri dish with *Fusarium oxysporum*-infected fungus (Ilk et al., 2017). Another study was conducted by Qian et al. (2011) where nanosized calcium carbonate carrying the active molecules was used to achieve slow release of the compounds. The effectiveness of validamycin loaded nanoparticles was inferior to validamycin alone against *Rhizoctonia solani* during first week of treatment, However, post two weeks the nanoparticle formulation demonstrated marginally superior outcomes compared to the active alone, emphasizing the longevity of the nanoformulation’s effectiveness. Kumar et al. (2017) observed that carbendazim-loaded polymeric nanoparticles exhibited increased rate of fungal inhibition against *Fusarium oxysporum* and *Aspergillus parasiticus* as compared to carbendazim alone. Phytotoxicity assays verified that the nanoformulated carbendazim had no adverse effect on plant germination and root development of *Zea mays*, *Lycopersicum esculentum*, and *Cucumis sativa* seeds. Santiago et al. (2019) reported that ChNp loaded with AgNP showed antibacterial activity against tomato bacterial wilt pathogen *Ralstonia solanacearum* and suggested AgNP entrapped chitosan is a suitable alternative to chemical antibiotics/bactericides.

Botanical extracts and essential oils having antimicrobial properties are most studied compounds in the era of organic farming. Several studies have been conducted to encapsulate the extracts and essential oils derived from plants to prevent its volatilization and to enhance the shelf life. Janatova et al. (2015) effectively encapsulated five distinct essential oil components into MSN and demonstrated greater antifungal activity 14 days post *Aspergillus niger* infection. Similar to this, SLNs have also been utilized to stabilize the essential oil of *Zataria multiflora*, offering defense against six fungi including soil-borne pathogen *Rhizoctonia solani* (Nasseri et al., 2016).

The major concern related to soil health is the leaching of chemicals through soil but limited studies have been conducted in this aspect. Wanyika (2013) encapsulated metalaxyl using MSNs and compared leaching in soil between encapsulated metalaxyl (11.5%) and free metalaxyl (76% release) for a period of 30 days. The relevance of conducting tests in a farming context was demonstrated by the encapsulated metalaxyl, which had a 47% higher release rate in water than in soil. Campos et al. (2015) investigated the cytotoxicity of carbendazim and/or tebuconazole placed onto two different types of nanoparticles, solid lipid or polymeric. In preosteoblast and fibroblast mouse cell lines, toxicity of the insecticides with nanoparticles was found to be reduced. Most of the studies have been conducted to develop nano-encapsulated fungicides however, the literature on encapsulation of antibiotics and antiviral agents against phytopathogens is rare. Thus, it indicates that more research in needed in this area.

### 4.3 Nanoparticles for induction of plant defense mechanism

#### 4.3.1 Antioxidant system

Plant health is disrupted severely by numerous soil-borne pathogens such as fungi, bacteria and viruses. In order to respond to vast array of biotic and abiotic stresses, plants have developed multifaceted defense systems which included both inducive and constitutive defenses. Constitutive mechanism presents first line of defense against the pathogen. Upon invasion of the pathogen into roots of plants bypassing constitutive defense mechanisms, leads to activation of induced defense mechanisms. For any management practices, induction of plant defenses is an important aspect. The production of ROS, which inhibits pathogen transmission and triggers local and systemic defense responses such as the release of pathogenesis-related (PR) proteins, is a component of the plant defense response to diverse stressors. Oxidative products are produced and the equilibrium between ROS and antioxidants is upset when the quantity of ROS exceeds the threshold. The antioxidant system in plants works to counteract the effects of oxidants. Superoxide dismutase (SOD), ascorbate peroxidase (APX), catalase (CAT), and guaiacol peroxidase (GPX) are a few of the enzymes that make up the antioxidant system (Tan et al., 2018). Nanoparticles alter cellular redox equilibrium by increasing or decreasing oxidative stress (Soares et al., 2018). According to previous studies, depending on the needs of the host plant, nanoparticles can either stimulate the generation of ROS, or suppress the oxidative burst by production of antioxidant enzymes and secondary metabolites. Abdelrhim et al. (2021) observed that Silicon dioxide nanoparticles (SiO_2_ NPs) activated antioxidant system and innate defense responses in wheat seedlings against the pathogen *R. solani*. SiO_2_ NP application increased the amount of photosynthetic pigments (chlorophylls and carotenoids), prompted the accumulation of defense-related stimulants (particularly salicylic acid), and reduced oxidative stress by activating both enzymatic (POD, SOD, APX, CAT, and PPO) and non-enzymatic (phenolics and flavonoids) antioxidant defense mechanisms. Plants exposed to nanoparticles showed increased expression of superoxide dismutase (SOD), which catalyzes the detoxification of O_2_ into either regular molecular oxygen (O_2_) or H_2_O_2_, and ascorbate peroxidase (APX), which detoxifies peroxides like H_2_O_2_ utilizing ascorbic acid (Asc) as a substrate (Fu et al., 2014). The enzymes that control the cellular Asc redox state, dehydroascorbate reductase (DHAR) and monodehydroascorbate reductase (MDAR), were downregulated (Fu et al., 2014). SOD, APX, and glutathione-S-transferase (GST) were found in greater abundance in AgNP treated *O. sativa* roots using proteomic analysis (Mirzajani et al., 2014). Additionally, these nanoparticles dramatically increased the activity of SOD and APX in *Pisum sativum* L. seedlings while inhibiting glutathione reductase (GR) and DHAR (Tripathi et al., 2017). When wheat roots were exposed to 500 mg/kg CuONPs, catalase (CAT), another enzyme that shields cells from oxidative damage, was noticeably increased (Dimkpa et al., 2012). When examined after 10 days, maize plants growing on soil supplemented with 0, 400, and 800 mg/kg Cerium dioxide nanoparticles (CeO_2_NPs) demonstrated a concentration-dependent increase in the buildup of H_2_O_2_ after 10 days, but 20th day showed no difference (Zhao et al., 2012). Lignin nanoparticle primed maize seeds showed positive effect on seed germination, radicle length in the initial stages. At later stages, increased biomass and biochemical parameters such as total soluble protein, total chlorophyll, carotenoid and anthocyanin were evident (Del Buono et al., 2021). Seed treatment of chickpeas with ChNPs exhibited increased germination percentage, biomass and seed vigor index (Saharan et al., 2013). ChNPs conjugated with rhizobacteria (PS2 and PS 10) showed increased seed germination, leaf area, plant height and chlorophyll content in maize plant. The stress tolerance mechanism in maize plant was attributed to greater production of antioxidant enzymes such as alkaline phosphatase, dehydrogenase and fluorescein diacetate hydrolysis (Khati et al., 2017). Abdelaziz et al. (2022) observed that ZnO-NPs prompted the healing of *F. oxysporum* infected eggplant by increasing morphological and metabolic markers such as plant height (152.5%), root length (106.6%), plant fresh biomass (146%), chlorophyll a (102.8%), chlorophyll b (67.86%), total soluble carbohydrates (48.5%), total soluble protein (81.8%), phenol (10.5%), antioxidant activity, and isozymes in comparison to infected control. It is becoming clear that the induction of antioxidant machinery by nanoparticles may foster plant growth as confirmed in a few investigations (Sharma et al., 2012; Burman et al., 2013; Kumar et al., 2013) as long as a harmful level of ROS is not reached in the cells. However, once this level is breached, this may result in impaired organ growth, development and induce phytotoxicity (Mittler, 2017).

#### 4.3.2 Phytohormones and plant signaling molecules

The signaling molecules salicylic acid (SA), Jasmonic acid (JA), and ethylene (ET) cause the proper defense reactions to be triggered. Gibberellin (GA), cytokinin (CK), auxin [indole-3-acetic acid (IAA)], abscisic acid (ABA), brassinosteroids (BRs), and strigolactone (SL) are other plant growth-regulating hormones that have the capacity to control defense responses Crosstalk between various plant hormones controls the balance between plant’s defenses and growth. Nanoparticles are known to affect the balance of plant hormones (Rastogi et al., 2017). Zahedi et al. (2019) reported accumulation of stress signaling molecules indole-3-acetic acid and abscisic acid, in strawberry plants treated with selenium nanoparticles (SeNPs). Additionally, increased levels of organic acids (such as malic, citric, and succinic acids) and sugars (such as glucose, fructose, and sucrose) in the fruits of strawberry plants treated with Se-NPs under saline conditions demonstrated the benefits of Se-NPs on the improvement of fruit quality and nutritional values. Azhar et al. (2021) examined phytohormone signaling when different metallic nanoparticles (ZnO, SiO2, and ZnO/SiO2 composite NPs) were exposed to Arabidopsis. They discovered that nanoparticle accumulation in plant tissue altered the expression level of genes associated with the cytokinin signalling pathway (ARR7 and ARR15), which suggested the significance of cytokinin in the plant’s response to nanoparticles. Shang et al. (2020) studied the effect of CuONPs on the soil-borne pathogen *Gibberella fujikuroi* that causes Bakanae disease in rice plants. They reported that seed treatment with copper sulphide nanoparticles significantly increased *in planta* JA content and shoot ABA content to levels equivalent to healthy control plants, while no difference in SA content was observed as compared to healthy and diseased control. Also, the level of sakuranetin (SN), an important phytoalexin in rice, was found to increase by 96.4% relative to diseased control when CuONPs were applied using foliar spray and seed treatment. Ma et al. (2021) studied the effect of nanoscale hydroxyapatite (nHA) on tomato plants infected with *Fusarium oxysporum* f.sp. *lycopersici.* They revealed that exposure to nHA significantly enhanced phenylalanine ammonialyase activity (30-80%) and total phenolic content (40-68%) in infected plants. The level of SA in shoots also increased by 10-45%, indicating a relationship between phytohormones and antioxidant pathways in nHA promoted defense against the fungal pathogen in the host plant. Another essential component of a plant’s immune system is PR proteins, which serve as a part of the diagnostic biomarkers of plant defense signaling pathways. The activation of the PR1, PR2, and PR5 genes indicates that the SA signaling pathway has increased (Ali et al., 2018). Healthy tobacco plants (*N. benthamiana*) when treated with SiO_2_ NPs and ZnONPs upregulated PR1 and PR2 genes that are SA-inducible, and treatment with magnetite nanoparticles (Fe_3_O_4_NPs) had a similar impact (Cai et al., 2020). As a result, the changed levels of phytohormones and PR proteins in plants exposed to nanoparticles suggest activation of the plant’s defense system. Grodetskaya et al. (2022) studied the effect of CuONPs on infection of downy birch micro-clones with soil-borne pathogens, *viz*., *Fusarium oxysporum* and *Fusarium avenaceum*, and assessed the level of expression of genes associated with defence responses in plants induced by microorganisms. CuONPs significantly suppressed the infection of *Fusarium avenaceum*, while no effect was observed against *Fusarium oxysporum.* Also, *a* decline in the expression of MYB46, PR-1, and PR-10 genes by 5.4 times was observed and could be due to a reduction in the pathogenic load caused by the effect of nanoparticles and the simultaneous stimulation of clones.”

## 5 Conclusion and future prospects

Soil borne plant pathogens represent diverse group of microorganisms such as fungi, bacteria, viruses, nematodes dwelling in soil and cause huge economic loss by affecting the root and collar region of plants. These pathogens are difficult to manage using conventional strategies as chemical pesticides can hardly reach into the soil system and large amount of these chemicals upsets the soil and environmental health. Nanotechnology has emerged as one of the potential management strategy to curb the menace of soil-borne plant pathogens. Nanomaterials can be effectively utilized to manage soil-borne pathogens owing to their versatile antimicrobial properties, including generation of ROS, cell membrane, organelles and other macromolecule destabilization and toxicity due to nanoparticles. The smaller size and greater surface area to volume ratio of nanoparticles allow for greater penetration potential and better interaction with soil-dwelling microbes thereby increasing their control efficacy. Nanomaterials can also be used as smart delivery vehicle for pesticides, fungicides and fertilizers to reduce the chemical load on the environment. Also, nanodiagnostics have emerged as a potential science to overcome the difficulties associated with the detection of soil-borne pathogens. Nanotechnology have been utilized to develop affordable biosensor systems for the early and sensitive detection of soil-borne pathogens based on the qualitative and quantitative detection of specific metabolites secreted by them. The indirect mechanism of management of soil-borne pathogens involves activation of plant defense mechanism and promotion of plant growth. To use nanomaterials wisely in soil-borne pathogen protection, it is imperative to understand their ecotoxicity, phytotoxicity, cytotoxicity, genotoxicity and interactions with the soil system and the soil residents. The nanomaterials upon reaching the soil, directly interact with soil particles affecting physicochemical properties, fertility and beneficial organisms. The effect of nanoparticles on soil structure, soil functioning, organic matter, siderophore production, nitrogen fixation, phosphate and potassium solubilization, and related processes need to be assessed to develop a holistic idea of the behaviour and fate of nanoparticles in soil. Also, the repercussions of the use of nanoparticles on beneficial microorganisms need to be examined. The mechanism involved in the interaction of nanoparticles with the rhizospheric microbiome needs to be elucidated further. Integration of nanomaterials with biological control agents and organic additives would prove beneficial in mitigating soil-borne diseases along with crop produce intensification. However, the technology is still in its infancy and demands extensive research in this arena. Further, nanocomposites can be developed using active ingredients and fertilizers for holistic development of agricultural crops.

Nanopesticides would require more than a decade to reach the market and its end users. Most of the nano-formulations or products are still in the laboratories or start-up phase and fewer products have reached the market till date. In 2019, the global market of nano-based materials was estimated as 8.5 billion US dollars and it was anticipated to flourish at an annual rate of 13.1% from 2020 to the year 2027 (Anonymous, 2016). The increase in market share of nanomaterials in different field is attributed to increase in social acceptance and demand and adoption in different arenas such as medical, food industry, agriculture, sports, aerospace, energy sectors and many more. However, acceptance of nano materials in agriculture is quite low as it is closely related to food and human health. The high initial production cost and complexity in production process makes its use in agriculture debatable. It is well known that nanomaterials synthesized through physical and chemical methods are enormously expensive and pose environmental risk. The green synthesis methods are comparatively cost effective and profitable in long run, less hazardous to the ecological health and have faster reaction rate. Efforts are made globally for regulating secure manufacturing and applications of nanomaterials and nanodevices by supervision and advices or by legislations (Choi et al., 2009). Till date, there is no single law fully committed to offer guidelines related to use of nanotechnology in any country across the globe. There is a need for guidelines and directives to assess impending hazards and for suggestions to ensure safe utilization of nanotechnology and few organizations are working actively in this field such as International Standard Organization (ISO), Organization for Economic Cooperation and Development (OCED) or US Food and Drug Administration (FDA) (Coles and Frewer, 2013). The cautious assessment of benefits of use of nanoparticles throughout its lifecycle addressing environmental, social and economic implications as well as occupational safety and health hazards are need of the hour. The effect of nanoparticles on agricultural, industrial and non-industrial workplaces and measurement of exposure of workers in the workplaces is important. Also, the toxicological properties of nanoparticles should be characterized and gathered information must be stored in database which can be readily accessed by researchers. In nanoparticle manufacturing plants, safety measures should be prioritized to eliminate occupational hazards. Also, safety guidelines should be established in laboratories concerning safe handling, use and disposal of nanoparticles and related waste materials. Proper care should be taken while promoting the benefits of use of nanoparticles in agricultural field so that no adversities result from their use.

Further research is needed to determine the practicability, sustainability, efficiency, applicability and releasability of nanotechnology-based products under field conditions as well as to validate these technologies in comparison to present technologies. Also, more *in vivo* and field trials are to be conducted for pesticide loaded nanomaterials or nanopesticides. To get comprehensive idea on efficacy of these pesticides, long term trial data is required which is constraint at present. Unlike chemical pesticides, nanomaterilals and nanopesticides lacks a clear definition by regulatory authorities. Kookana et al. (2014) reviewed into great detail about how the effects of nanopesticides, in contrast to conventional pesticides, may rely on the uptake, bioavailability, concentration, and toxicity of the nanoparticles as well as the ratio of the active coupled to them. There is also little information available on the problem of pesticide resistance and potential ways that adding nanoparticles could lower its prevalence. The creation of regulatory standards for risk assessment is not possible without the use of extensive analytical techniques. The application of pesticides in field undergoes rotation and revision by regulatory bodies periodically restricts and ban the use of chemical pesticides thus, broad range of nanoformulations need to be available for future applications. Using new tools and methods to produce solid data for analysis, characterization, and risk assessment may be the key to receiving approval from regulatory authorities.

Thus, for logical selection of appropriate nanomaterials and nanopesticides, a thorough knowledge of the structural characteristics of the nanoparticles, including their shape, size, functional groups, and active adsorption/loading capacity is essential. In order to undertake biocompatibility and efficacy investigations at the cell, organism, and pest-host ecosystem levels under as-close-to-field circumstances as feasible, it is also crucial to choose a trustworthy and reproducible system. Further, wholesome development of nanoscience requires integration of different sciences such as biologists, agricultural engineers, plant pathologists, biotechnologists and soil microbiologists. Therefore, efforts must be directed toward creating a soil disease control strategy that is long-lasting, safe, effective, and environmentally benign.

## Author contributions

PD was responsible for writing guidance and manuscript preparation. AK was responsible for writing the first draft of the article. PD revised the article with other coauthors. All authors contributed to the article and approved the submitted version.
